# Pathways of Epithelial Barrier Breakdown and Remodeling in Esophageal Organoids by Commonly Used Surfactants

**DOI:** 10.1111/all.70490

**Published:** 2026-08-27

**Authors:** Ozge Ardicli, Duygu Yazici, Huseyn Babayev, Yagiz Pat, Manal Bel Imam, Hang Du, Hassan Melhem, Carina Beha, Laura Buergi, Anja Heider, Juan‐Felipe López, Sena Ardicli, Can Zeyneloğlu, Andrea Kreienbühl, Nino Stocker, Patrick Westermann, Christoph B. Messner, Mübeccel Akdis, Jan Hendrik Niess, Alain Schoepfer, Luc Biedermann, Cezmi A. Akdis, Swiss EoE Cohort Study Group, Patrick Aepli, Luc Biedermann, Ruggero Biral, Carine Blanchard, Lorenzo Botteselle, Simon Buetikofer, Emanuel Burri, Joachim Diebold, Annika Eckhold, Annett Franke, Michèle Frei, Thomas Greuter, Chantal Hasler, Wolfram Jochum, Tanja Kilchmann, Seraina Koller, Andrea Kreienbühl, Fritz Murray, Peter Netzer, Gabrielle Reichhart, Elodie Ristorcelli, Gerhard Rogler, Jean‐Benoit Rossel, Ekaterina Safroneeva, Catherine Saner, Christoph Schlag, Jon‐Duri Senn, Alain Schoepfer, Philipp Schreiner, Christine Sempoux, Dagmar Simon, Hans‐Uwe Simon, Alex Straumann, Sven Trelle, Achim Weber, Niels Willi, Marcel Zwahlen, Willem van de Veen

**Affiliations:** ^1^ Swiss Institute of Allergy and Asthma Research (SIAF) University of Zurich Davos Switzerland; ^2^ Division of Food Processing, Milk and Dairy Products Technology Program, Karacabey Vocational School Bursa Uludag University Bursa Türkiye; ^3^ Gastroenterology Group, Department of Biomedicine University of Basel Basel Switzerland; ^4^ Department of Genetics, Faculty of Veterinary Medicine Bursa Uludag University Bursa Türkiye; ^5^ Department of Gastroenterology and Hepatology University Hospital Zurich Zurich Switzerland; ^6^ Department of Gastroenterology and Hepatology University Digestive Healthcare Center, Clarunis Basel Switzerland; ^7^ Division of Gastroenterology and Hepatology, Centre Hospitalier Universitaire Vaudois University of Lausanne Lausanne Switzerland

**Keywords:** cocamidopropyl betaine, eosinophilic esophagitis, epithelial barrier dysfunction, esophageal organoids, sodium dodecyl sulfate

## Abstract

**Background:**

Eosinophilic esophagitis (EoE) is a chronic immune‐mediated disease characterized by symptoms of esophageal dysfunction, such as dysphagia and food impaction, as well as eosinophilic inflammation and impaired epithelial barrier integrity, which promotes allergen influx. Environmental exposures that weaken the esophageal barrier, including surfactants found in personal care products such as sodium dodecyl sulfate (SDS) and cocamidopropyl betaine (CAPB), have been proposed as contributors to disease susceptibility. However, their direct effects on esophageal epithelial barrier function and associated molecular responses remain insufficiently characterized.

**Methods:**

We investigated the molecular and functional consequences of SDS and CAPB exposure in primary EoE‐derived esophageal organoids and air‐liquid interface cultures derived from biopsy tissue samples. We conducted transepithelial electrical resistance (TEER) measurements, paracellular flux assays, RNA sequencing, targeted and untargeted proteomics, and immunofluorescence imaging.

**Results:**

Treatment of esophageal organoids with SDS or CAPB (0–100 μg/mL) caused dose‐dependent cytotoxicity and barrier disruption, with SDS producing stronger effects. At 12.5 μg/mL, viability declined to ~30% with SDS versus ~60% with CAPB, accompanied by greater TEER reduction and ~2‐fold higher permeability. RNA‐seq of organoids exposed to 12.5 μg/mL for 24 h showed distinct transcriptomic clustering, with 2749 shared and > 1500 surfactant‐specific differentially expressed genes, indicating broad epithelial remodeling, cell‐cycle suppression, and stress responses, more pronounced under SDS. SDS preferentially activated inflammatory and innate immune pathways, which was supported by proteomic detection of elevated cytokines (TNF, IL‐8, IL‐18, etc.). Both surfactants induced keratinization, epidermal cell differentiation, and partial epithelial‐mesenchymal transition signatures, including increased vimentin without E‐cadherin loss.

**Conclusion:**

These findings demonstrate that common surfactants can induce coordinated epithelial stress responses in esophageal tissue models and may act as environmental aggravating factors in EoE at relatively low doses. This work underscores the need to consider mucosal exposure to consumer product ingredients in the context of epithelial barrier disorders, particularly surfactants such as SDS and CAPB, and supports efforts to refine formulations to minimize epithelial damage.

## Graphical abstract

This study shows that SDS and CAPB directly impair esophageal epithelial barrier integrity in primary EoE‐derived models. SDS induces stronger inflammatory, oxidative stress, and keratinization responses than CAPB. Both surfactants promote epithelial remodeling and partial epithelial‐mesenchymal transition (EMT), with CAPB showing a distinct extracellular matrix remodeling profile. Common surfactants in personal care products may act as environmental aggravating factors in eosinophilic esophagitis. ALI, air‐liquid interface; CAPB, cocamidopropyl betaine; CCL20, C‐C motif chemokine ligand 20; EoE, Eosinophilic esophagitis; GLRX, glutaredoxin‐1; IL, interleukin; KRT80, keratin 80; NPX, normalized protein expression; MCP‐1, monocyte chemoattractant protein‐1; RNA‐seq, RNA sequencing; SDS, sodium dodecyl sulfate; SIRT2, sirtuin 2; TEER, transepithelial electrical resistance; TNF, tumor necrosis factor; TXNDC5, thioredoxin domain containing 5.

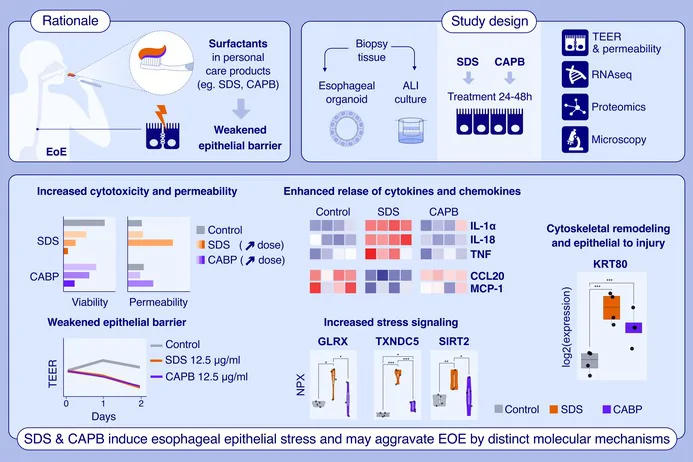

## 1 Introduction

Eosinophilic esophagitis (EoE) is a chronic inflammatory disease characterized by dense eosinophilic infiltration of the esophageal mucosa and associated tissue remodeling, including epithelial hyperplasia, lamina propria fibrosis, and architectural distortion [1, 2, 3]. A defining pathogenic feature of EoE is epithelial barrier dysfunction, which contributes to both disease initiation and progression [4, 5]. Under physiological conditions, the mucosal barrier forms the first line of defense against dietary and environmental antigens. When this barrier is disrupted, tissue permeability increases, facilitating immune activation and inflammation [6, 7, 8]. Consequently, EoE is increasingly conceptualized as a *barrier disease*, shaped by genetic susceptibility, allergic and inflammatory triggers, and epithelial vulnerability [8, 9, 10].

Recent research has highlighted environmental exposures as critical modulators of epithelial integrity [11, 12, 13, 14]. Among these, surfactants used in household and personal care products, including toothpaste, mouthwash, shampoos, and dish detergents, have attracted attention because of their membrane‐active and irritant properties [15, 16, 17]. Sodium dodecyl sulfate (SDS), a widely used anionic surfactant, can solubilize lipids and proteins in cellular membranes, disrupt tight junctions, generate structural discontinuities, and increase paracellular permeability, thereby facilitating allergen penetration and type 2 immune responses [11, 13]. Experimental models show that SDS exposure induces hallmark EoE‐like features, including barrier breakdown and eosinophilic inflammation [18].

Cocamidopropyl betaine (CAPB) is a zwitterionic surfactant derived from coconut oil and marketed as a milder alternative to SDS in products labeled as “sensitive skin” or “hypoallergenic” [19, 20]. Although CAPB has reduced lipid‐extracting capacity compared to SDS, chronic exposure or high concentrations can still provoke irritant or allergenic responses [17]. To date, its effect on the esophageal epithelium in the context of EoE has not been systematically examined.

Recent studies indicate that these exposures can alter esophageal microbiome composition and epithelial gene expression, producing histological and molecular changes consistent with EoE [18, 21, 22]. In this context, repeated oral exposure to surfactants such as SDS and CAPB, even at low concentrations present in consumer products, may compromise epithelial defenses and promote allergic sensitization. These findings raise public health concerns about the long‐term safety of these ubiquitous surfactants, particularly in individuals with genetic or immunological predispositions.

Here, we investigated the effects of SDS and CAPB on three‐dimensional esophageal epithelial spheroids (hereafter referred to as organoids) and air‐liquid interface (ALI) cultures. We employed an integrated multi‐modal approach combining transepithelial electrical resistance (TEER) measurements, FITC‐dextran paracellular flux assays, immunofluorescence imaging, and transcriptomic and proteomic profiling to assess how these surfactants influence epithelial integrity, inflammatory signaling, differentiation, and stress‐response pathways. Specifically, we sought to (1) compare the epithelial barrier‐disruptive potential of SDS and CAPB; (2) delineate the molecular programs induced by each compound, focusing on inflammation, keratinization, and epithelial‐mesenchymal transition (EMT); and (3) identify regulatory mechanisms that may exacerbate or mitigate epithelial injury caused by surfactant exposure in the context of EoE.

## 2 Materials and Methods

### 2.1 Human Subjects

Esophageal epithelial cells were isolated from four individuals (*n* = 4) with confirmed EoE. All patients were in an inactive phase of EoE at the time of sampling. This selection was intended to minimize confounding effects of active eosinophilic inflammation and to allow assessment of epithelial‐intrinsic responses to surfactant exposure. The details about the cohort are shown in Table S1. EoE patients were recruited at the EoE Clinics of the University Hospital Zurich (USZ) with informed consent obtained from all participants and were part of the Swiss EoE Cohort Study (SEECS), a multicentric prospective database study (BASEC Number PB_2016‐01962). Biopsies were collected during routine endoscopic evaluations at the Division of Gastroenterology and Hepatology, University Hospital Zurich, Switzerland. The study was approved by the local ethics committee (EKNZ 2015‐388). Diagnosis was based on ≥ 15 eosinophils per high‐power field and symptoms of esophageal dysfunction (e.g., solid food dysphagia, food impaction, odynophagia, heartburn, vomiting, or abdominal pain). None of the patients showed evidence of gastroesophageal reflux disease (GERD).

### 2.2 EoE Primary Cell Isolation and Organoid Culture

Esophageal epithelial organoids were generated from EoE biopsies following a modified protocol from Kaymak et al. [23] Biopsies were collected in Keratinocyte‐serum‐free‐medium‐c (KSFM; Gibco, Thermo Fisher Scientific, Waltham, MA, USA, 17005042) supplemented with 50 μg/mL bovine pituitary extract (BPE; Gibco), 1 ng/mL epidermal growth factor (EGF; Gibco), 100 U/mL penicillin, 100 μg/mL streptomycin (Sigma Aldrich, Taufkirchen, Germany, P4333), 100 μg/mL kanamycin sulfate (Gibco, 15160‐054), 0.5 μg/mL amphotericin B (Gibco, 2556000A), and 0.09 mM calcium chloride (Sigma‐Aldrich, 10043‐52‐4). Samples were processed on the day of collection to preserve cell viability.

Tissues were digested using 10 U/mL dispase I (Corning, NY, USA, 354235) for 10 min at room temperature (RT), followed by washing with phosphate‐buffered saline (PBS). Subsequently, tissues were subjected to further dissociation in 0.05% trypsin–EDTA (Gibco, 15400‐054) at 37°C for 10 min at 800 rpm using an Eppendorf ThermoMixer C. Cells were washed with KSFM‐c medium and filtered sequentially through 70 and 35 μm cell strainers, centrifuged at 300 × *g* for 5 min at 4°C, and counted.

A total of 20,000–30,000 cells were resuspended in 40 μL of Cultrex Basement Membrane Extract (BME), Type 2, Pathclear (R&D Systems), per dome. Droplets were polymerized at 37°C for 25 min, followed by the addition of KSFM‐c (0.6 mM Ca^2+^), supplemented with 10 μM ROCK inhibitor Y27632 (Tocris). Y27632 was excluded from subsequent medium changes (every other day). Organoids were cultured for 11 days at 37°C in a 5% CO_2_ incubator. Surfactant treatment was initiated on Day 11; organoids were maintained for an additional 24–48 h, depending on the downstream analysis performed (Figure 1A).

**FIGURE 1:**
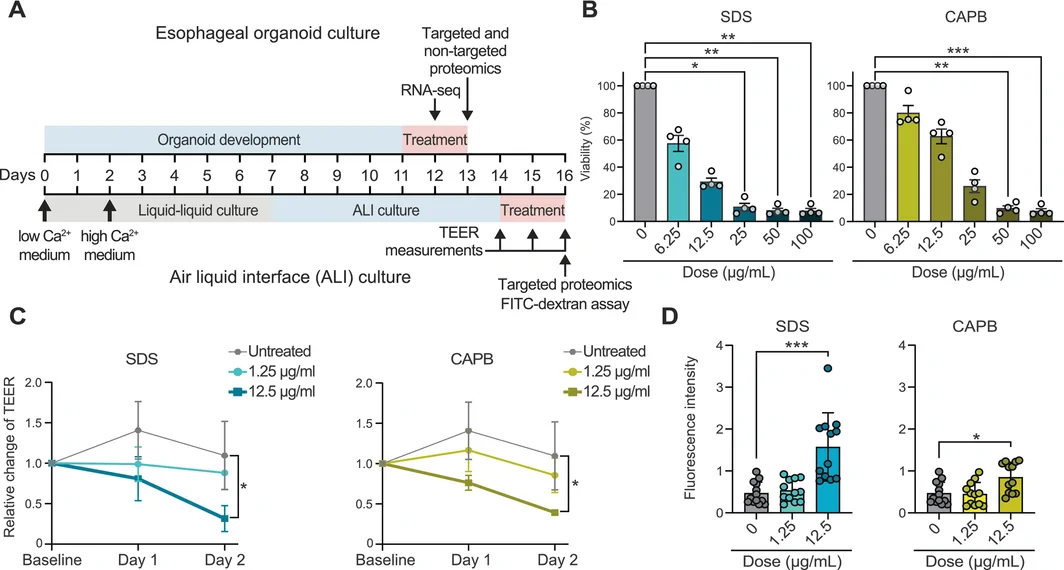
Effects of SDS and CAPB on esophageal organoid viability and barrier function. (A) Experimental workflow for esophageal organoid and ALI cultures. Organoids were expanded in low‐ and high‐calcium media, followed by surfactant treatment and complementary transcriptomic and proteomic profiling. ALI cultures were used for TEER measurements and FITC‐dextran permeability assays. Organoids on Days 12 and 13 indicate 24 and 48 h posttreatment endpoints, respectively. For ALI cultures, surfactant exposure was initiated after 14 days of culture, and functional readouts were collected during the following 48 h. (B) Organoid viability after 24 h exposure to increasing concentrations of SDS or CAPB, measured by MTT assay. Statistical testing: Kruskal–Wallis test with Dunn's multiple‐comparison correction versus untreated control; *p < 0.05, **p < 0.01, ***p < 0.001. (C) TEER measurements in ALI cultures during 48 h exposure to 1.25 or 12.5 μg/mL SDS or CAPB (*n* = 4 donors, 3–4 replicates). Statistical analysis: Kruskal–Wallis test with Dunn's multiple‐comparison correction versus untreated control; *p < 0.05. (D) FITC‐dextran flux in ALI cultures during 48 h exposure to 1.25 or 12.5 μg/mL SDS or CAPB (*n* = 4 donors, 3 replicates). Statistical testing: Kruskal–Wallis test with Dunn's multiple‐comparison correction; *p < 0.05, ***p < 0.001.

### 2.3 Air‐Liquid Interface Culture

Esophageal epithelial cells expanded in monolayer culture (KSFM‐c, 0.09 mM Ca^2+^) were seeded at 150,000 cells per 0.5 cm^2^ onto 24‐well transwell inserts with 0.4 μm polyester membranes (Oxyphen, Wetzikon, Switzerland). After 2 days, the medium was switched to high‐calcium KSFM‐c (Ca^2+^ 1.8 mM), and the medium was replaced every other day until Day 7. On Day 7, the apical medium was removed to establish ALI, and basal medium was supplemented with 10 ng/mL KGF/FGF7 (R&D Systems) and 75 μg/mL L‐ascorbic acid (Sigma‐Aldrich). Cells were cultured for a total of 14 days prior to surfactant exposure, including an initial submerged culture phase followed by differentiation under ALI conditions from Day 7 onward. After surfactant treatment, cultures were maintained for an additional 48 h for TEER and FITC‐dextran analyses (Figure 1A).

### 2.4 Surfactant Preparation and Exposure Protocol

SDS and CAPB (≥ 99% purity, Sigma‐Aldrich) were dissolved in sterile PBS and stored at 4°C. Working solutions were freshly prepared. Based on cytotoxicity assays, 12.5 μg/mL was selected for downstream mechanistic experiments to enable comparison of epithelial responses under matched exposure conditions. Each experiment included untreated controls, SDS‐treated, and CAPB‐treated groups.

### 2.5 Cytotoxicity Assay

Organoid viability was assessed using the MTT assay (Invitrogen, Oregon, USA) following the manufacturer's instructions. A total of 3000 single cells per dome were embedded in 5 μL BME in 96‐well plates (one dome per well). Organoids were treated on Day 11 with increasing concentrations of the surfactant (0, 6.25, 12.5, 25, 50, and 100 μg/mL). After 24 h, MTT reagent was added and incubated at 37°C for 4 h: the resulting formazan was solubilized in DMSO. Absorbance was measured at 540–570 nm using a BioTek plate reader.

### 2.6 TEER Measurement

TEER was measured using an Epithelial Voltohmmeter (EVOM) (Millicell ERS‐2, Millipore, USA) with specialized chopstick electrodes designed for 24‐well transwell inserts. During surfactant exposure, TEER values were recorded at baseline, 24 h, and 48 h posttreatment and were normalized to the insert surface area.

### 2.7 Paracellular Flux Assay

Paracellular permeability was measured using FITC‐dextran (3–5 kDa; Sigma‐Aldrich). After 14 days of ALI culture, cells were exposed to SDS or CAPB (1.25 and 12.5 μg/mL) for 48 h. FITC‐dextran (400 μL) was applied to the apical surface, and fluorescence was measured after 4 h using a BioTek plate reader.

### 2.8 RNA Extraction From Organoids

Following treatment, organoids were washed with PBS and recovered from the basement membrane matrix using Cell Recovery Solution (Corning) at 4°C for 30 min. Organoids were then centrifuged and the pellet was lysed in RLT buffer (Qiagen RNeasy Mini Kit, Cat. No: 74034) containing 1% β‐mercaptoethanol. Lysates were vortexed and stored at −80°C until RNA extraction. RNA was isolated using the Qiagen RNeasy Mini Kit (Cat. No: 74034) according to the manufacturer's instructions and quality confirmed using an Agilent Bioanalyzer (RIN > 7.0).

### 2.9 RNA‐Sequencing (RNA‐Seq) Analysis

The library was constructed using poly (A) selection and sequenced on an Illumina NovaSeq 6000 platform, yielding ~30 million paired‐end reads per sample. Raw sequencing reads were quality‐trimmed and filtered using BBDuk (BBTools suite) and aligned to the human genome (GRCh38) with Subread aligner. Gene counts were subsequently quantified using featureCounts. Given the heterogeneity inherent in primary patient‐derived organoids, we applied the ComBat‐seq algorithm (sva package) to correct for donor‐specific batch effects. ComBat‐seq uses a negative binomial regression model to adjust for technical and biological noise attributed to the donor source while retaining the integer nature of the data required for downstream RNA‐seq tools. Differential expression analysis was performed on the adjusted count matrix using DESeq2 (Wald test). Pathway enrichment analyses employed Gene Set Enrichment Analysis (GSEA) and Over Representation Analysis (ORA) to reveal overrepresented biological functions or canonical pathways. Genes of particular interest included those related to inflammation, EMT, keratinization, tight junction integrity, and autophagy.

### 2.10 Targeted Proteomics

Secreted proteins in 48 h organoid supernatants were analyzed using Olink Target‐96 panels (Inflammation, Metabolism, Organ Damage; Olink, Uppsala, Sweden). Protein levels were quantified as log2‐scaled Normalized Protein eXpression (NPX) values. Statistical analysis was performed using the OlinkAnalyze R package (v3.9) via a Linear Mixed‐Effects Model (LMM), with condition as a fixed effect and donor as a random effect. Pairwise post hoc comparisons were adjusted for multiple testing using the Benjamini‐Hochberg method (FDR < 0.05).

### 2.11 Untargeted Proteomics

Untargeted proteomics were conducted on organoid lysates. After PBS washes, samples were incubated in 500 μL Cell Recovery Solution (Corning) at 4°C for 30 min, centrifuged (250 × *g*, 3 min), and the pellet was lysed in RIPA buffer (Merck, Cat. No. 20‐188) supplemented with protease inhibitors (Roche, Cat. No. 4693116001). Lysates were vortexed and incubated at 4°C for 30 min, then stored at −80°C. Protein lysates were processed using the same automated SP3 (autoSP3) sample preparation workflow described previously [24]. Briefly, proteins were reduced, alkylated, captured on mixed hydrophilic/hydrophobic magnetic beads, and subjected to on‐bead digestion with Trypsin/Lys‐C. Peptides were acidified, dried, reconstituted, quantified, and loaded onto Evotips according to the manufacturer's instructions. Liquid chromatography was performed using an Evosep One system, and peptides were analyzed on a ZenoTOF 7600 mass spectrometer (SCIEX) operating in data‐independent acquisition (DIA) mode with the Scanning SWATH workflow. Raw data were processed in Spectronaut v19 (Biognosys AG, Switzerland) using the directDIA+ workflow and searched against the *Homo sapiens* UniProt reference proteome. Protein identification and quantification were performed using Spectronaut's integrated false discovery rate (FDR) control, and only proteotypic peptides were retained for downstream analyses.

### 2.12 Confocal Microscopy and Epithelial‐Mesenchymal Transition Marker Analysis

For immunofluorescence analyses, organoids were exposed to SDS or CAPB for 72 h prior to fixation and staining. Organoids were fixed in 4% paraformaldehyde, permeabilized with 0.1% triton X‐100, blocked with 1% BSA, and stained with primary antibodies against E‐cadherin and vimentin (VIM). Alexa Fluor‐conjugated secondary antibodies and DAPI nuclear counterstain were applied (Table S2). Samples were mounted with antifade reagent, and images were acquired on a Zeiss LSM780 confocal microscope under standardized settings.

### 2.13 Statistical Analyses

Data were analyzed with GraphPad Prism 10 or R (v4.5.0). Normally distributed data were evaluated by one‐way ANOVA with Tukey's post hoc test. Nonparametric data were analyzed by Mann–Whitney *U* or Kruskal–Wallis with Dunn's correction. RNA‐seq and proteomics data were adjusted for multiple testing using Benjamini‐Hochberg FDR. Statistical significance was set at *p* < 0.05.

## 3 Results

### 3.1 SDS Induces More Pronounced Cytotoxicity and Barrier Disruption in Esophageal Organoids Compared to CAPB

The experimental workflow involved establishing esophageal epithelial organoids as well as differentiating the cells into ALI cultures, followed by exposure to increasing concentrations of SDS or CAPB to assess viability and barrier function (Figure 1A). Figure S1 provides representative microscopic images illustrating organoid development on Days 3, 5, 7, 9, and 11, as well as morphological changes after 24 and 48 h of exposure to 12.5 μg/mL SDS or 12.5 μg/mL CAPB on Days 12 and 13. To examine the cytotoxic potential of SDS and CAPB, we treated esophageal organoids with increasing concentrations (0–100 μg/mL) and measured viability using the MTT assay (Figure 1B). Both SDS and CAPB reduced viability in a dose‐dependent manner. At a concentration of 12.5 μg/mL (1:80,000 w/v), SDS reduced organoid viability to 29.17%, while CAPB produced a much milder effect, decreasing the viability to only 62.63%, demonstrating a clear difference in their cytotoxic potential. Higher concentrations of both surfactants resulted in further significant decreases in cell viability.

In ALI cultures, relative TEER fell significantly after exposure to both surfactants at a 12.5 μg/mL dose, with a larger and progressive decline under SDS. Specifically, SDS reduced TEER by Day 1 and further by Day 2, whereas CAPB produced a smaller but significant reduction over the same period; untreated cultures remained stable (Figure 1C). FITC‐dextran permeability assays confirmed increased paracellular flux, with SDS‐treated epithelia showing ~2× higher permeability than CAPB at 12.5 μg/mL (Figure 1D). These data confirm that both surfactants impair epithelial barrier integrity, with SDS exerting a stronger disruptive effect. However, particularly for SDS at 12.5 μg/mL, the observed TEER reduction and increased FITC‐dextran permeability should be interpreted as the combined consequence of epithelial injury, reduced viable epithelial cell mass, and barrier remodeling rather than as evidence of remodeling alone. At the lower concentration tested in ALI cultures (1.25 μg/mL), TEER and FITC‐dextran permeability were not significantly altered, supporting a dose‐dependent barrier‐injury response. These findings demonstrate that barrier impairment was dose‐dependent, with significant functional disruption observed only at the higher surfactant concentration.

### 3.2 SDS and CAPB Drive Overlapping and Unique Gene Expression Profiles Involving Differentiation, Immune Activation, and Cell Cycle Suppression

To characterize the molecular responses underlying surfactant‐induced epithelial injury, we performed bulk RNA‐seq on organoids treated with SDS or CAPB (12.5 μg/mL for 24 h). Principal component analysis (PCA) demonstrated clear segregation of untreated, SDS‐ and CAPB‐treated samples, with PC1 and PC2 accounting for 72% and 10% of the total variance, respectively (Figure 2A). A sample correlation matrix further confirmed that SDS‐ and CAPB‐treated organoids clustered apart from untreated controls (Figure 2B), consistent with distinct transcriptomic reprogramming. Differential expression analysis revealed extensive transcriptional reprogramming following both treatments, with 2749 genes regulated in common, and 1703 and 1543 uniquely altered by SDS and CAPB, respectively (Figure 2C). Among the shared genes, 1474 were upregulated and 1263 downregulated, whereas SDS‐unique genes included 992 upregulated and 711 downregulated, and CAPB‐unique genes included 618 upregulated and 925 downregulated (Table S3). A clustered heatmap (Figure 2D) illustrates these distinct transcriptional signatures, showing that both surfactants triggered widespread remodeling, with SDS eliciting broader and more pronounced changes.

**FIGURE 2:**
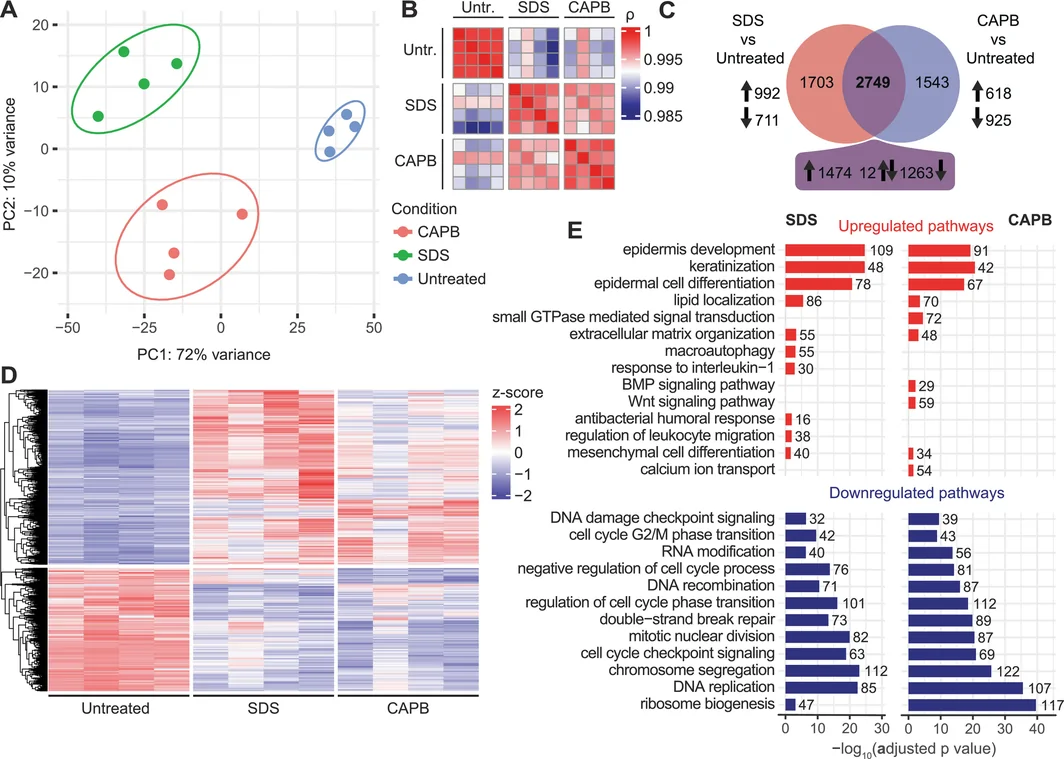
Transcriptomic profiling of SDS‐ and CAPB‐treated esophageal organoids. (A) PCA plot of global gene expression profiles from untreated, SDS (12.5 μg/mL)‐, and CAPB (12.5 μg/mL)‐treated samples. PC1 and PC2 explain 72% and 10% of the variance. (B) Correlation matrix of the top 500 most highly expressed genes across conditions, showing within‐condition clustering. (C) Venn diagram of significantly regulated DEGs (adjusted *p* < 0.05) in SDS 12.5 μg/mL vs. untreated and CAPB 12.5 μg/mL vs. untreated organoids. Upward arrows (↑) indicate upregulated genes, whereas downward arrows (↓) represent downregulated genes. Gene counts marked with both arrows (↑↓) indicate those that are upregulated in one condition and downregulated in the other. (D) Heatmap (*z*‐score normalized) of selected DEGs, showing distinct expression patterns across conditions. Differential expression was assessed using DESeq2 with Benjamini‐Hochberg FDR correction. The genes displayed in the heatmap are listed in Table S3. (E) Overrepresentation analysis of up‐ and downregulated pathways in SDS‐ and CAPB‐treated organoids, showing pathway enrichment based on FDR‐adjusted *p* values.

Pathway enrichment analyses showed that both surfactants upregulated pathways associated with epithelial remodeling, including epidermal development, keratinization, epidermal cell differentiation, and lipid localization, although the magnitude of enrichment differed between treatments (Figure 2E). SDS uniquely enriched pathways involved in macroautophagy, interleukin‐1 signaling, leukocyte migration, and antibacterial responses, reflecting a stronger innate immune activation. CAPB, by contrast, more prominently enriched Wnt, calcium ion transport, BMP signaling pathways, and small GTPase‐mediated signaling. Downregulated pathways overlapped across conditions, including suppression of cell‐cycle regulation, chromosome segregation, DNA replication, and ribosome biogenesis. CAPB produced a broader suppression of translational machinery and mitotic progression (e.g., 117 genes in ribosome biogenesis versus 47 for SDS). Taken together, both surfactants alter differentiation and immune response‐related transcriptional programs, while engaging partly distinct pathways.

### 3.3 SDS Induces Upregulation of Inflammatory Responses in Esophageal Organoids

We next examined the expression levels of key inflammatory and epithelial stress‐related genes following treatment with SDS and CAPB. Some inflammasome‐associated genes, including *PYCARD* and *NLRP1*, were downregulated after SDS treatment, whereas CAPB‐treated organoids exhibited expression levels comparable to those of untreated controls (Figure 3A). The damage‐associated molecular pattern (DAMP)‐related gene *HMGB1* and the chemokine receptor *CXCR2* were also decreased under both treatments, dampening upstream danger sensing and chemotactic signaling. In contrast, multiple epithelial inflammatory mediators were induced, forming a stress‐associated transcriptional signature. Both surfactants increased expression of *MEFV*, *IL36B*, *S100A8*, *S100A9*, *RELA*, *IL1RN*, *NLRP2*, and *STAT3*, representing a core epithelial stress module. SDS specifically induced the expression of *IL36G*, *IL1B*, *CXCL8* (*IL8*), and *MMP25*, genes associated with cytokine release, neutrophil recruitment, and extracellular matrix (ECM) remodeling.

**FIGURE 3:**
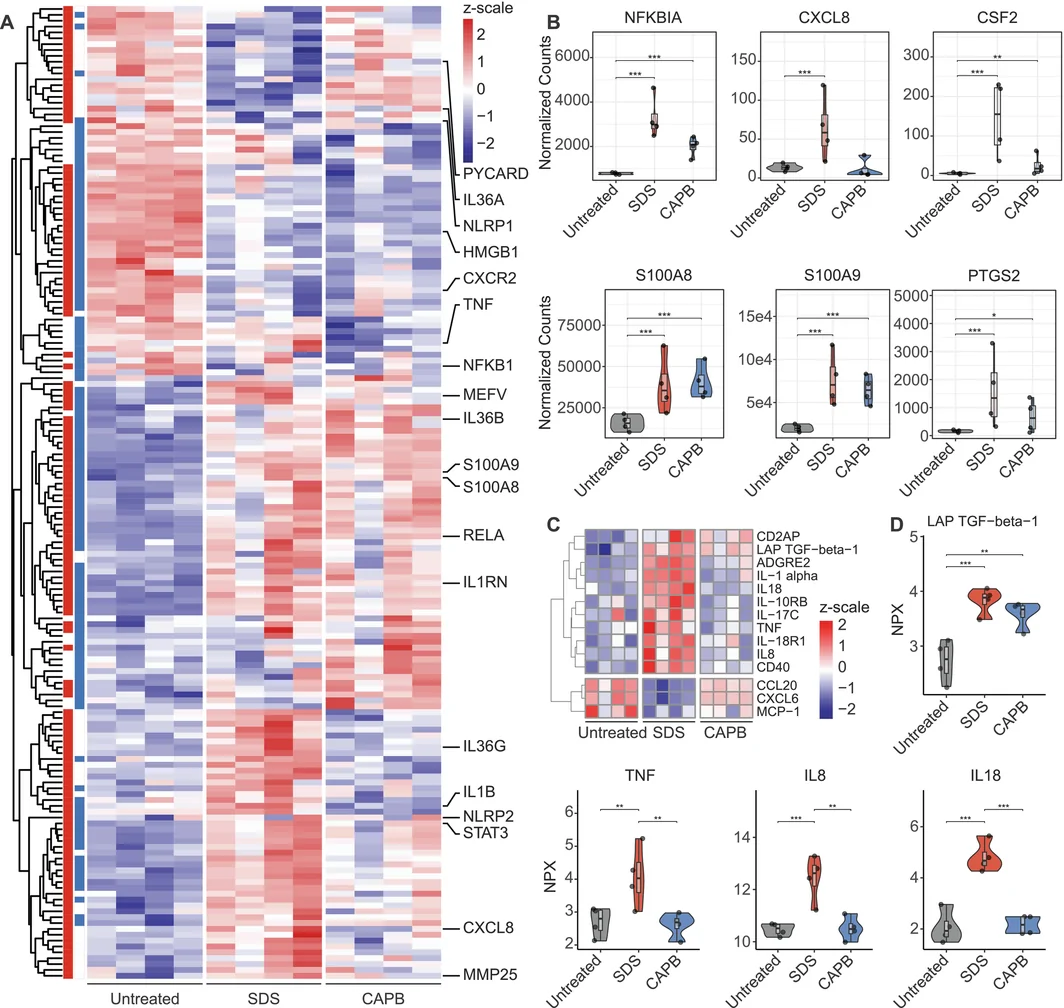
SDS induces robust inflammatory transcriptional and proteomic responses in esophageal organoids. (A) Heatmap (*z*‐score normalized) of inflammation‐ and epithelial‐related transcripts from RNA‐seq analysis of untreated, SDS‐treated (12.5 μg/mL), and CAPB‐treated (12.5 μg/mL) organoids. Statistical significance for RNA‐seq transcript plots reflects DESeq2 adjusted *p* values. (B) Violin plots of selected transcripts (*NFKBIA, CXCL8, CSF2, S100A8, S100A9, PTGS2*) from RNA‐seq data across conditions. (C) Heatmap (*z*‐score normalized) of inflammatory proteins from Olink proteomics (NPX values) showing protein‐level responses to SDS and CAPB. (D) Violin plots of selected inflammatory mediators (TNF, IL‐8, LAP TGF‐β1, IL‐18) based on Olink NPX values. Olink NPX values were analyzed using a linear mixed‐effects model with donor as a random effect and Benjamini‐Hochberg FDR correction; *p < 0.05, **p < 0.01, ***p < 0.001.

SDS exposure markedly upregulated *NFKBIA*, a central regulator of the NF‐κB signaling pathway, relative to untreated controls (*p* < 0.001; Figure 3B), consistent with robust NF‐κB pathway activation. This pattern was mirrored by *CXCL8* (*p* < 0.001), and the growth factor *CSF2* (GM‐CSF), which showed a robust SDS‐induced increase (*p* < 0.001) and a smaller but significant rise under CAPB (*p* < 0.01). RNA‐seq results indicated that the calcium‐binding stress markers *S100A8* and *S100A9*, which are key mediators of epithelial defense and neutrophil recruitment, were dramatically upregulated by SDS (*p* < 0.001) and significantly elevated by CAPB (*p* < 0.001). Similarly, *PTGS2* (COX2), an enzyme central to prostaglandin synthesis, was strongly induced by SDS (*p* < 0.001) and modestly by CAPB (*p* < 0.05; Figure 3B).

Targeted proteomics profiling further confirmed the enhanced release of cytokines and chemokines (Figure 3C). Heatmap analysis revealed broad induction of inflammatory mediators, with SDS producing a stronger and more extensive upregulation across the panel. SDS markedly elevated TNF, IL‐8, and IL‐18 (Figure 3D), alongside increases in IL‐1α, IL‐17C, and CD40. CAPB exposure led to a more moderate induction profile. Conversely, SDS reduced levels of MCP‐1 (CCL2), CCL20, and CXCL6 relative to both untreated and CAPB, suggesting a selective attenuation of chemokine‐driven leukocyte recruitment. This selective attenuation, together with *NFKBIA* upregulation and suppression of cell‐cycle and ribosome biogenesis pathways, indicates a stress‐adapted epithelial state that releases pro‐inflammatory cytokines while limiting broad immune cell trafficking.

Both SDS and CAPB significantly increased LAP TGF‐β1 secretion (*p* < 0.001; Figure 3D) to a comparable extent, consistent with epithelial wound‐healing responses. Overall, SDS elicited a stronger pro‐inflammatory cytokine response than CAPB, aligning with transcriptomic evidence of more pronounced innate immune activation.

### 3.4 Differential Oxidative‐Stress Responses Distinguish SDS and CAPB Exposure

Figure 4A illustrates the expression patterns of oxidative‐stress‐related genes derived from bulk RNA‐seq, revealing distinct clustering patterns across untreated, SDS‐ and CAPB‐exposed organoids. SDS induced a broad transcriptional activation of peroxiredoxins, thioredoxin‐associated genes, and NRF2‐dependent antioxidant regulators, whereas CAPB triggered a comparatively modest and variable response. In contrast, the corresponding proteomic NPX data highlight only a subset of oxidative stress‐related proteins, with concordant protein‐level changes. Notably, SDS markedly increased levels of GLRX, TXNDC5, SIRT2, and NCF2 (markers for redox balance and stress signaling) while CAPB exhibited minimal or no measurable protein induction (Figure 4B).

**FIGURE 4:**
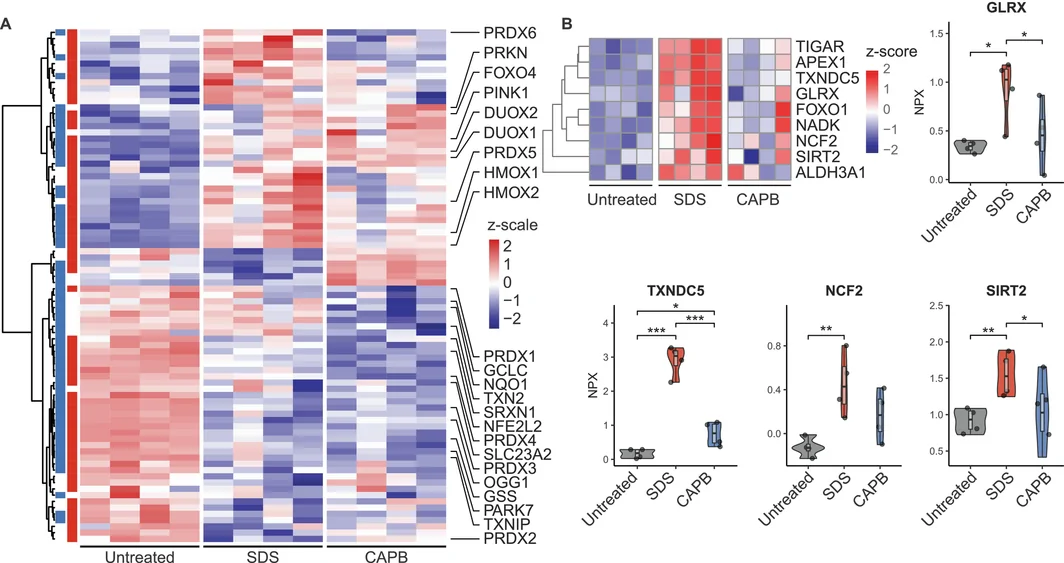
Transcriptomic and proteomic profiling of oxidative stress‐related genes and proteins. (A) Heatmap of RNA‐seq‐derived expression patterns of antioxidant and redox‐regulating transcripts across untreated, SDS‐treated (12.5 μg/mL), and CAPB‐treated (12.5 μg/mL) organoids. (B) Heatmap and violin plots showing protein‐level changes for selected targets. Olink NPX values were analyzed using a linear mixed‐effects model with donor as a random effect and Benjamini‐Hochberg FDR correction; *p < 0.05, **p < 0.01, ***p < 0.001.

### 3.5 Surfactant Exposure Induces Epithelial Cell Keratinization and Differentiation Responses at the Transcriptomic and Proteomic Levels

Because epithelial keratinization and terminal differentiation represent canonical stress‐adaptive responses to skin barrier injury [24], we next examined whether surfactant exposure triggers these remodeling pathways in esophageal organoids. Hierarchical clustering and heatmap analysis revealed robust upregulation of keratinization and epidermal differentiation‐related genes following exposure to SDS and CAPB (Figure 5A). This shift was further supported by suppression of *TP63* together with modulation of differentiation‐associated keratins such as *KRT10*, indicating a transition from basal to differentiated epithelial states. Moreover, the increased expression of cornified envelope components such as *IVL* suggests activation of a stress‐associated keratinization program rather than physiological epithelial differentiation. Both surfactants upregulated a shared cluster of cornified envelope and desmosomal genes (*DSP*, *KRT16*, *CDSN*, *DSG4*, *SPRR1A/B*, *SPRR2G*, *SPRR3*, *TGM1*, *IVL*, and *NFKBIZ*) (Figure 5A,B). SDS caused particularly strong induction of *LORICRIN*, suggesting intensified activation of terminal envelope formation under increased barrier stress. However, it simultaneously suppressed mature barrier components such as *FLG* and *KRT10*, indicating partial loss of structural integrity despite increased differentiation signaling (Figure 5A,B). CAPB, while triggering a similar differentiation program, uniquely increased *FLG* and reduced *EREG* (encodes a protein that is involved in a wide range of biological processes, including inflammation, wound healing, and cell proliferation), and *GRHL2* (a critical transcription factor for epithelial development), suggesting a more balanced response that partially preserves barrier integrity while attenuating growth factor and polarity‐associated transcriptional programs.

**FIGURE 5:**
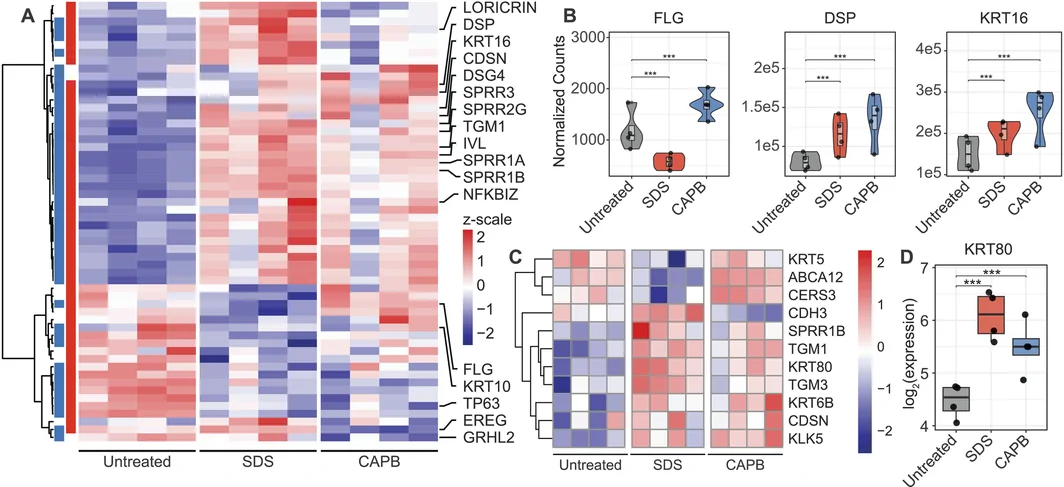
Surfactant exposure alters keratinization‐associated transcripts and proteins in esophageal organoids. (A) Heatmap (*z*‐score normalized) of keratinization‐associated transcripts from RNA‐seq analysis of untreated, SDS‐treated (12.5 μg/mL), and CAPB‐treated (12.5 μg/mL) organoids. Statistical significance for RNA‐seq transcript plots reflects DESeq2 adjusted *p* values. (B) Violin plots of selected transcripts (*FLG, DSP, KRT16*) showing normalized RNA‐seq counts across conditions. (C) Heatmap (*z*‐score normalized) of keratinization‐ and differentiation‐related proteins from untargeted proteomics across untreated, SDS‐treated, and CAPB‐treated organoids. Differential protein abundance was assessed from untargeted proteomics data using FDR‐adjusted *p* values. (D) Violin plot of KRT80 protein abundance from untargeted proteomics. ***p < 0.001.

Untargeted proteomics provided complementary insights (Figure 5C). Basal epithelial and lipid homeostasis proteins, including CERS3, ABCA12, and KRT5, were markedly decreased after SDS exposure compared with both untreated and CAPB‐treated organoids, suggesting disruption of ceramide synthesis, lipid transport, and basal keratinocyte structure. CAPB, in contrast, maintained or modestly increased expression of these proteins, consistent with a milder effect on epidermal homeostasis. Proteins involved in terminal differentiation and cornified envelope formation, TGM1, TGM3, SPRR1B, and CDSN, were elevated under both SDS and CAPB, confirming transcriptional trends.

A key stress‐induced cytoskeletal marker, KRT80, was significantly upregulated by both surfactants (Figure 5D), indicating cytoskeletal remodeling and epithelial adaptation to injury. Collectively, these findings demonstrate that surfactant exposure triggers coordinated transcriptional and proteomic changes consistent with epithelial remodeling, characterized by the induction of keratinization programs, suppression of basal and lipid barrier components, and activation of terminal differentiation pathways. SDS exerts a stronger and more disruptive effect, whereas CAPB induces a controlled differentiation response compatible with partial barrier preservation.

### 3.6 Surfactant Exposure Induces Epithelial‐Mesenchymal Transition‐Associated Pathways in Esophageal Organoids

Because epithelial injury can trigger plasticity programs reminiscent of EMT, we next examined whether SDS and CAPB influence EMT‐related signaling, cytoskeletal remodeling, and extracellular matrix regulation. Both surfactants altered the expression of multiple genes associated with epithelial plasticity and tissue remodeling, including *BMP2*, *TGFB1*, *CTNNB1*, *FGFR1*, *EPB41L5*, *S100A4*, and *SOX9*, while reducing the expression of several epithelial‐associated regulators, including *NOTCH1*, *SLC39A6*, and *SMAD4* (Figure 6A).

**FIGURE 6:**
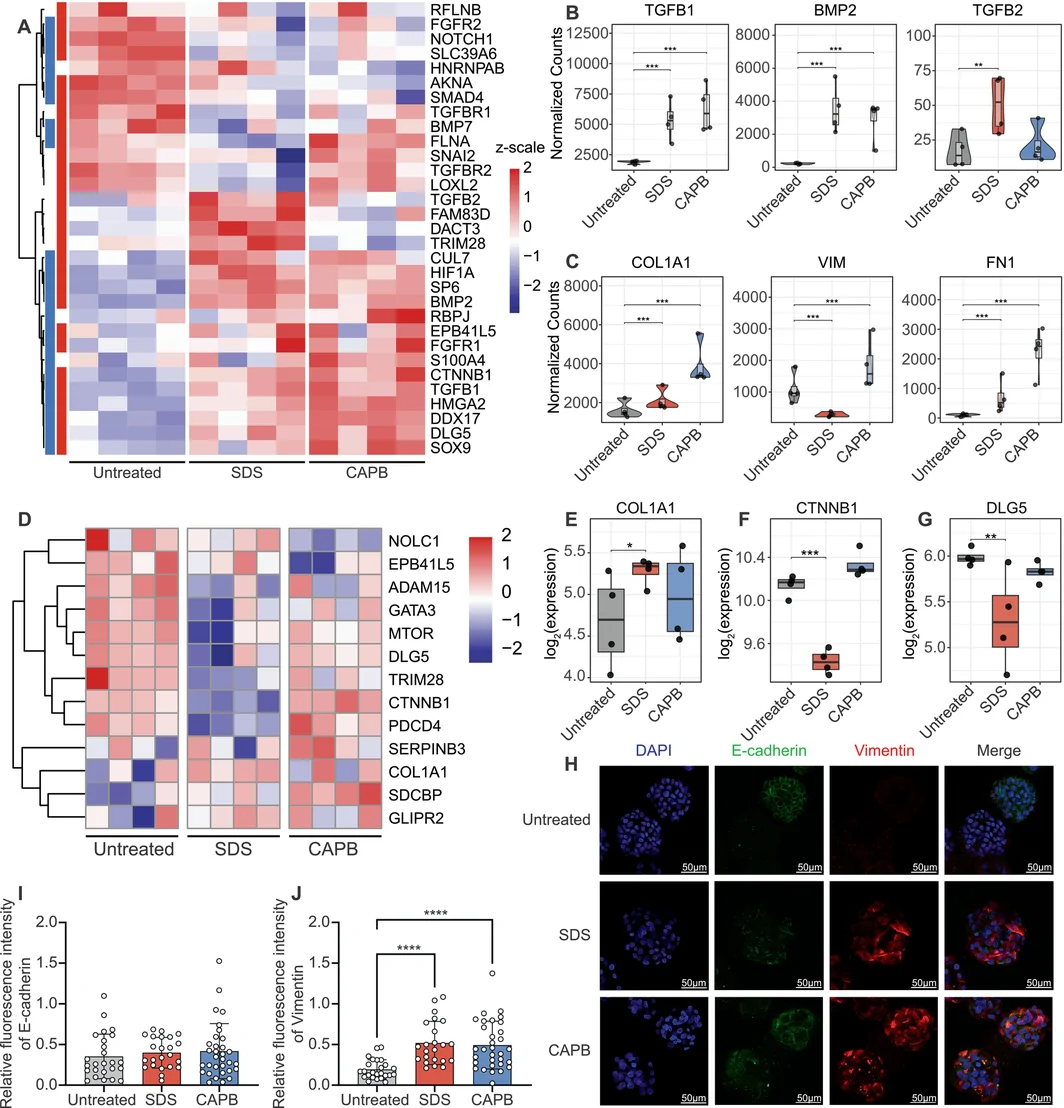
SDS and CAPB induce EMT‐associated transcriptional, proteomic, and imaging changes in esophageal organoids. (A) Heatmap (*z*‐score normalized) of EMT‐associated transcripts from RNA‐seq analysis of untreated, SDS‐treated (12.5 μg/mL), and CAPB‐treated (12.5 μg/mL) organoids. (B, C) Violin plots of selected EMT‐related transcripts (*TGFB1, BMP2, TGFB2, COL1A1, FN1, VIM*) showing normalized counts across conditions. Statistical significance for RNA‐seq transcript plots reflects DESeq2 adjusted *p* values. (D) Heatmap (*z*‐score normalized) of EMT‐ and adhesion‐related proteins from untargeted proteomics in untreated, SDS‐treated, and CAPB‐treated organoids. Differential protein abundance was assessed from untargeted proteomics data using FDR‐adjusted *p* values. (E–G) Violin plots of selected EMT‐related proteins (e.g., COL1A1, CTNNB1, DLG5) showing log2‐transformed expression values. (H) Immunofluorescence staining for DAPI (nuclei), E‐cadherin, and vimentin (VIM) in untreated, SDS‐treated, and CAPB‐treated organoids after 72 h of surfactant exposure. (I, J) Quantification of relative fluorescence intensity of E‐cadherin (I) and VIM (J). Immunofluorescence quantification was analyzed using Kruskal–Wallis test with Dunn's multiple‐comparison correction; ****p < 0.0001.

Both surfactants significantly upregulated *TGFB1* and *BMP2* compared with untreated controls (*p* < 0.001; Figure 6B). CAPB displayed a slightly higher median *TGFB1* expression, consistent with robust TGF‐β pathway engagement, while *TGFB2* increased selectively under SDS (*p* < 0.01; Figure 6B), suggesting differential regulation of TGF‐β isoforms depending on surfactant type. *BMP2* showed a similar pattern, indicating engagement of the BMP axis in response to surfactant exposure. Analysis of mesenchymal and extracellular‐matrix markers (Figure 6C) showed strong upregulation of *COL1A1* and *FN1* in both conditions (*p* < 0.001), whereas *VIM* expression diverged, suppressed by SDS (*p* < 0.001) but elevated by CAPB (*p* < 0.001). Thus, CAPB promoted a transcriptional profile consistent with matrix remodeling and mesenchymal traits, while SDS induced a more stress‐repressive phenotype. Of note, these transcriptomic data were obtained after 24 h of surfactant exposure, whereas VIM protein expression was assessed by immunofluorescence after 72 h. Thus, the reduced *VIM* transcript levels observed in SDS‐treated organoids at 24 h and the increased VIM protein detected at the later timepoint may reflect timepoint‐dependent regulation during epithelial stress and remodeling.

Untargeted proteomics revealed additional differences in structural and signaling‐related proteins (Figure 6D). SDS broadly reduced expression of key regulators, including MTOR (mechanistic target of rapamycin, a central regulator of cell growth, metabolism, protein synthesis, and autophagy), CTNNB1 (β‐catenin, playing a role in cell proliferation and adhesion by linking cadherins to the actin cytoskeleton), GATA3 (a major type 2 transcription factor), and the polarity‐associated protein DLG5. In contrast, CAPB largely preserved or modestly increased several proteins, such as CTNNB1 and SERPINB3 (a regulator in pathways related to autocrine and paracrine signaling, cellular protein metabolism, and the innate immune system), and showed a less pronounced reduction in MTOR. These patterns suggest that SDS more strongly suppresses epithelial growth and Wnt/β‐catenin signaling, whereas CAPB exerts milder effects. Consistent with this, significant changes in COL1A1 (Figure 6E), CTNNB1 (Figure 6F), and DLG5 (Figure 6G) were observed in SDS‐treated organoids but not in CAPB‐treated samples.

Immunofluorescence imaging (Figure 6H–J) was performed to determine whether these transcriptional and proteomic changes were translated into altered epithelial architecture over time. In untreated organoids, E‐cadherin displayed strong membranous localization with minimal VIM, consistent with an intact epithelial phenotype. Following SDS or CAPB exposure, VIM expression increased markedly, whereas E‐cadherin localization remained largely preserved. Quantitative analysis confirmed these findings: both surfactants significantly elevated VIM levels compared with controls (*p* < 0.0001), while E‐cadherin expression remained stable. In short, these results indicate a partial EMT response, characterized by induction of mesenchymal markers and cytoskeletal remodeling without full loss of epithelial identity.

## 4 Discussion

SDS and CAPB are widely used in liquid dishwashers, toothpastes, mouthwashes, and cosmetic products, creating the potential for repeated contact with esophageal mucosa [25]. CAPB is generally regarded as a milder alternative to anionic surfactants [26], yet clinical and experimental reports demonstrate that it can still provoke irritation and delayed‐type hypersensitivity reactions in susceptible individuals [20, 27, 28, 29]. SDS, by contrast, is well‐established as a potent irritant with strong lipid‐solubilizing capacity and the ability to disrupt tight junctions and membrane structure [11, 12, 13, 14, 18]. These properties raise particular concern for barrier‐vulnerable tissues such as the esophageal epithelium, especially in individuals with underlying epithelial fragility, including patients with EoE. Despite their broad use, the comparative effects of SDS and CAPB on esophageal epithelial biology have not been systematically evaluated. In this study, we used primary EoE‐derived organoids and ALI cultures to provide a comprehensive transcriptomic and proteomic characterization of epithelial responses to these surfactants.

Here, we demonstrate that both surfactants activate broad epithelial stress programs, but SDS elicits substantially stronger and more disruptive responses across functional assays, transcriptomics, and proteomics. CAPB triggered overlapping pathways, including inflammatory signaling, keratinization, and cytoskeletal remodeling, yet the magnitude of these effects was consistently lower. These findings refine the common perception of CAPB's relative safety and suggest that, although less potent than SDS, it is not biologically inert, particularly in the context of pre‐existing conditions with barrier vulnerability and with possible synergisms with other barrier‐damaging substances, such as food emulsifiers [30], carbonated and sugary drinks [31], micro‐ and nanoplastics, alcohol, and several protease allergens [32].

Notably, the present study was performed using organoids derived from patients with histologically inactive EoE. This experimental design allowed us to investigate the direct epithelial responses to SDS and CAPB while minimizing the confounding effects of active eosinophilic inflammation. Therefore, the observed molecular and functional alterations are more likely to reflect epithelial‐intrinsic responses to surfactant exposure rather than secondary consequences of ongoing inflammatory activity. Although this experimental design minimizes the confounding effects of active eosinophilic inflammation, it does not determine whether surfactant‐induced epithelial responses differ between inactive EoE, active EoE, and healthy esophageal tissue.

Our complementary transcriptomic and proteomic analyses revealed coordinated activation of inflammatory, keratinization‐associated, and epithelial plasticity programs following surfactant exposure. Although these pathways were engaged by both compounds, SDS consistently produced stronger transcriptional and proteomic effects, reflecting more profound epithelial stress. CAPB activated similar pathways but at a lower magnitude, suggesting a qualitatively comparable but quantitatively attenuated injury response.

Moreover, SDS exposure was associated with changes consistent with temporally evolving epithelial injury responses. Although the present study was not designed as a formal time‐course experiment, the use of distinct endpoints for RNA‐seq, proteomics, and immunofluorescence allows indirect comparison of early transcriptional responses and later protein‐level or structural epithelial changes. These findings should therefore be interpreted as suggestive of temporally evolving epithelial injury responses, rather than as a direct longitudinal time‐course. At 24 h, early transcriptional changes included suppression of *VIM* together with upregulation of keratinization pathways and transient maintenance of barrier‐associated proteins such as *DLG5* and *CTNNB1*. By 48 h of SDS exposure, proteomic analyses revealed declines in DLG5 and CTNNB1, indicating an emerging loss of adhesion and barrier support. In the separate 72 h immunofluorescence experiment, VIM expression rose markedly, consistent with a shift toward cytoskeletal remodeling and partial EMT. Together, these findings suggest that SDS does not provoke a single acute injury response but may drive a staged transition from early stress adaptation and keratinization to later structural compromise and epithelial plasticity.

The apparent divergence between *VIM* transcript abundance at 24 h and VIM protein expression after 72 h highlights the dynamic nature of epithelial stress responses. Transcriptomic analyses may capture an early phase of acute stress adaptation, whereas later protein‐level changes may reflect delayed translation, altered protein turnover, or progressive cytoskeletal remodeling. Because these measurements were obtained from distinct experimental endpoints rather than a formal time‐course experiment, they should be interpreted as consistent with, rather than definitive proof of, time‐dependent epithelial remodeling.

These dynamics may have implications for real‐world exposure. SDS can persist in the oral mucosa and esophageal surface during and after toothbrushing, resulting in sustained mucosal contact [21]. Although our organoid model involves continuous exposure of epithelial cells to surfactants, which differs from the transient exposure in vivo, several factors may increase contact time in patients. In EoE, esophageal stasis due to fibrostenosis (e.g., food impaction or subobstruction), motility disturbances, or both can prolong mucosal exposure. In addition, concentrations in consumer products (1%–10%) far exceed those tolerated by esophageal organoids [18]. A complementary study by Wright et al. (submitted) reports that SDS exposure during toothbrushing results in measurable esophageal exposure and barrier alterations in humans, supporting the physiological relevance of our findings [33].

Thus, the early‐to‐late progression observed in vitro may reflect a continuum of epithelial adaptation that could occur in vivo under repeated or sustained exposure. The cumulative nature of these responses underscores the importance of evaluating surfactant safety. The epithelial remodeling signatures we observed following SDS and CAPB exposure are consistent with models of chronic epithelial stress in barrier‐mediated diseases [8]. Both surfactants induced keratinization pathways characteristic of an activated squamous epithelium, including upregulation of *KRT16*, SPRR family members, and additional cornified envelope proteins. These alterations reflect a shift toward a high‐turnover, injury‐responsive state [34] that attempts to reinforce the barrier but also compromises normal tissue organization.

Our findings also demonstrate that surfactant exposure can initiate partial EMT‐like programs, marked by VIM induction, upregulation of extracellular matrix genes (including *COL1A1* and *FN1*), and cytoskeletal reorganization, while maintaining core epithelial identity such as E‐cadherin expression. This pattern aligns with the concept of partial EMT, a reversible epithelial plasticity state increasingly recognized in barrier diseases. Although complete EMT is rare in nonmalignant esophageal epithelium [35], partial EMT can weaken barrier integrity, enhance antigen penetration, and amplify inflammatory signaling, processes central to EoE pathophysiology.

Differences in remodeling trajectories between SDS and CAPB were particularly notable. CAPB‐treated organoids showed pronounced upregulation of extracellular matrix genes (*COL1A1*, *FN1*) and robust VIM induction, together with partial preservation of epithelial polarity and Wnt/β‐catenin signaling markers such as CTNNB1 and SERPINB3 (untargeted proteomics, 48 h). This pattern suggests an adaptive remodeling phenotype that maintains elements of epithelial integrity despite stress exposure. In contrast, SDS triggered an early keratinization‐dominant response, followed by the suppression of polarity proteins (CTNNB1, DLG5), and later, VIM induction and cytoskeletal reorganization, indicating a more destabilizing remodeling trajectory.

In addition, SDS strongly induced a range of NF‐κB‐dependent cytokines and epithelial stress mediators, including IL8, IL18, *CSF2*, *S100A8*, *S100A9*, and *PTGS2*, alongside keratinization markers characteristic of activated squamous epithelium. CAPB elicited qualitatively similar but attenuated inflammatory responses. These patterns indicate that surfactants do not induce a single injury axis but rather generate a composite inflammatory‐stress state that closely resembles the epithelial phenotypes observed in EoE.

Together, these observations converge on a central theme: surfactant exposure does not simply provoke acute irritation, but engages multiple interacting injury pathways, including keratinization, inflammatory activation, cytoskeletal remodeling, extracellular matrix deposition, and partial EMT that are closely related to the pathophysiology of EoE. The degree and balance of these responses differ between surfactants, with SDS driving more intense and destabilizing changes, whereas CAPB still exerts tissue‐damaging effects that may be problematic, particularly in individuals with pre‐existing conditions of epithelial fragility. These findings support the plausibility of SDS and CAPB as environmental aggravating factors in EoE and underscore the importance of evaluating surfactant exposure in disease prevention and management.

The implications for EoE pathogenesis are substantial. Epithelial barrier fragility, stress‐induced keratinization, inflammatory mediator release, and partial EMT are hallmarks of EoE biology [36, 37]. Our data show that surfactant exposure alone can amplify these processes. SDS is used at concentrations ranging from 0.5% to 3% in toothpaste [18, 38], levels far exceeding those tolerated by organoids; yet, even at lower doses, it provoked robust inflammatory, structural, and differentiation changes, raising concerns about cumulative effects. CAPB, though milder, induced ECM remodeling and mesenchymal features that may contribute to fibrosis or altered barrier function over time [39]. Thus, SDS and CAPB differ in magnitude, but not in principle, as both can engage pathways relevant to chronic epithelial remodeling and barrier vulnerability in EoE.

The concentration used in the present study (12.5 μg/mL; 0.00125% w/v) is substantially lower than the SDS concentrations typically found in commercial oral care products. While SDS and CAPB are both commonly used surfactants in toothpaste formulations, they are not necessarily incorporated at identical concentrations, and CAPB concentrations vary considerably across products and are often not publicly disclosed by manufacturers. Therefore, the selected concentration was intended to model a biologically relevant epithelial exposure rather than to replicate the formulation concentration of either surfactant in consumer products.

Our findings also highlight the value of a complementary transcriptomic and proteomic approach. Integration of RNA‐seq, targeted and untargeted proteomics, and functional readouts (TEER, FITC‐dextran flux, imaging) provided a coherent mechanistic framework linking surfactant exposure to epithelial injury, differentiation, and remodeling. This approach improves our understanding of how common chemical exposures interact with epithelial biology to shape disease‐relevant phenotypes.

Several limitations warrant consideration in the present study. Organoids lack immune and stromal compartments, restricting observations to epithelial‐intrinsic responses. Future work should incorporate co‐culture or organ‐on‐chip models to capture epithelial‐immune‐fibroblast interactions. Additionally, real‐world SDS concentrations exceed the tolerable range for esophageal organoids, preventing direct high‐dose testing. Nonetheless, this limitation underscores potential risk, as in vivo exposures may elicit even stronger effects. Dose–response studies, repeated‐exposure models, and evaluation of protective interventions (e.g., antioxidants, barrier‐enhancing agents) are needed to refine risk assessment. Another limitation of the present study is that viability was assessed in organoids, whereas TEER and FITC‐dextran permeability were measured in ALI cultures. Therefore, although the data support dose‐dependent surfactant‐induced barrier impairment, the current experiments cannot fully separate epithelial cell loss from junctional remodeling in the ALI model. Future studies incorporating matched cytotoxicity and viability measurements in ALI cultures, particularly under repeated low‐dose exposure conditions, will be important to define the threshold at which reversible barrier dysfunction progresses to overt epithelial injury.

Using complementary transcriptomic, proteomic, and functional analyses, we demonstrate that SDS and CAPB provoke distinct but overlapping injury responses in primary EoE organoids. SDS induces profound epithelial disruption, inflammatory activation, keratinization, partial EMT‐associated remodeling, and suppression of proliferative programs. CAPB elicits milder but still substantial transcriptional and proteomic changes, including ECM remodeling and mesenchymal features that may exacerbate epithelial vulnerability over time. These findings highlight the potential of common surfactants in personal care products to aggravate epithelial injury, and underscore the value of reformulating consumer products to minimize mucosal irritation. Continued integration of patient‐derived organoids in remission or exacerbation with in vivo and clinical studies will be essential to translate these insights into preventive and therapeutic strategies. An additional important area for future investigation will be the direct comparison of surfactant‐induced responses in healthy donor‐derived and EoE‐derived esophageal epithelial models. Such studies may help determine whether EoE‐derived epithelium exhibits disease‐specific susceptibility to environmental surfactants or a more general epithelial stress response.

## Author Contributions

W.V. conceived and designed the study. O.A., D.Y., H.B., Y.P., M.B.I., H.D., H.M., C.B., L.B., A.H., J.‐F.L., S.A., C.Z., N.S., P.W., and C.B.M. performed experiments and/or acquired data. A.K., A.S., and L.B. contributed to patient recruitment, clinical data acquisition, and sample collection. O.A., D.Y., H.B., Y.P., P.W., and C.B.M. analyzed and interpreted the data. W.V., C.A.A., M.A., L.B., and J.H.N. provided critical input, supervision, and resources. O.A. and W.V. drafted the manuscript. All authors revised the manuscript critically for important intellectual content and approved the final version.

## Funding

This study was supported by the Promedica Stiftung (Switzerland) (grant no. 1515/M to W. van de Veen), and by the Swiss National Science Foundation (SNSF) (grant 310030_219210 to J.H.N.), SNSF (grant no. 32003B_204751/1 to A. M. Schoepfer).

## Conflicts of Interest

A.S. has received consulting fees from AstraZeneca, BMS‐Receptos, Calypso, EsoCap, Falk Pharma, GSK, Pfizer, Sanofi‐Regeneron, and Shire. L.B. has received advisory board fees from AbbVie, Amgen, BMS, Falk, Janssen, Pfizer, Lilly, Takeda, Sanofi, and EsoCap, and speaker fees from Takeda, Sanofi, AbbVie, Lilly, Falk, BMS, and Pfizer. W.V. has received research grants from the Promedica Stiftung, the Swiss EoE Foundation, and the Novartis Research Foundation. C.A.A. has received research grants from the Swiss National Science Foundation, the European Union, AO Research Institute Davos, Novartis Research Institutes, Stanford University, Harvard University, Seed Health, and SciBase; serves on advisory boards for Sanofi/Regeneron, Stanford University Sean Parker Asthma Allergy Center, Novartis, GlaxoSmithKline, Bristol‐Myers Squibb, Seed Health, and SciBase; and is Editor‐in‐Chief of Allergy. M.A. has received research grants from the Swiss National Science Foundation, the European Union, the Leading House for the Latin American Region, and the Sean N. Parker Center for Allergy Research at Stanford University, and serves on advisory boards for the Leo Foundation Skin Immunology Research Center and the Stanford University Sean Parker Asthma Allergy Center. All other authors declare no conflicts of interest. None of the disclosed relationships are directly related to the present work.

## Supporting information


**Figure S1:** Representative phase‐contrast images of organoids during development. Phase‐contrast microscopy images of organoid development captured on Days 3, 5, 7, 9, and 11, as well as Days 12 and 13 of culture following 24 and 48 h of surfactant treatment. The baseline corresponds to Day 11, with treatment images shown for Day 12, which is considered Day 1 of the 2‐day exposure period. Morphological changes following treatment with 12.5 μg/mL SDS or 12.5 μg/mL CAPB are also shown.

**Table S1:** Baseline demographic, clinical, endoscopic, histopathological, and treatment characteristics of the patients.
**Table S2:** Information on the primary and secondary antibodies used in confocal microscopy.

**Table S3:** Differentially expressed genes (DEGs) in esophageal organoids following surfactant exposure. Columns show independent deregulation status (Up, Down, NS), log2 fold change (LFC), and adjusted *p*‐value (*p*adj) for both treatments against untreated controls (FDR < 0.05, LFC > 1).

## Data Availability

The data that support the findings of this study are available from the corresponding author upon reasonable request.
