# Supplementary material for: Pathways of Epithelial Barrier Breakdown and Remodeling in Esophageal Organoids by Commonly Used Surfactants
Source: Allergy. 2026 Aug 27;81(10):3659–72. doi: 10.1111/all.70490 (PMC13646889; doi:10.1111/all.70490)
Supplement: Supplementary file 1 [file ALL-81-3659-s002.pdf]

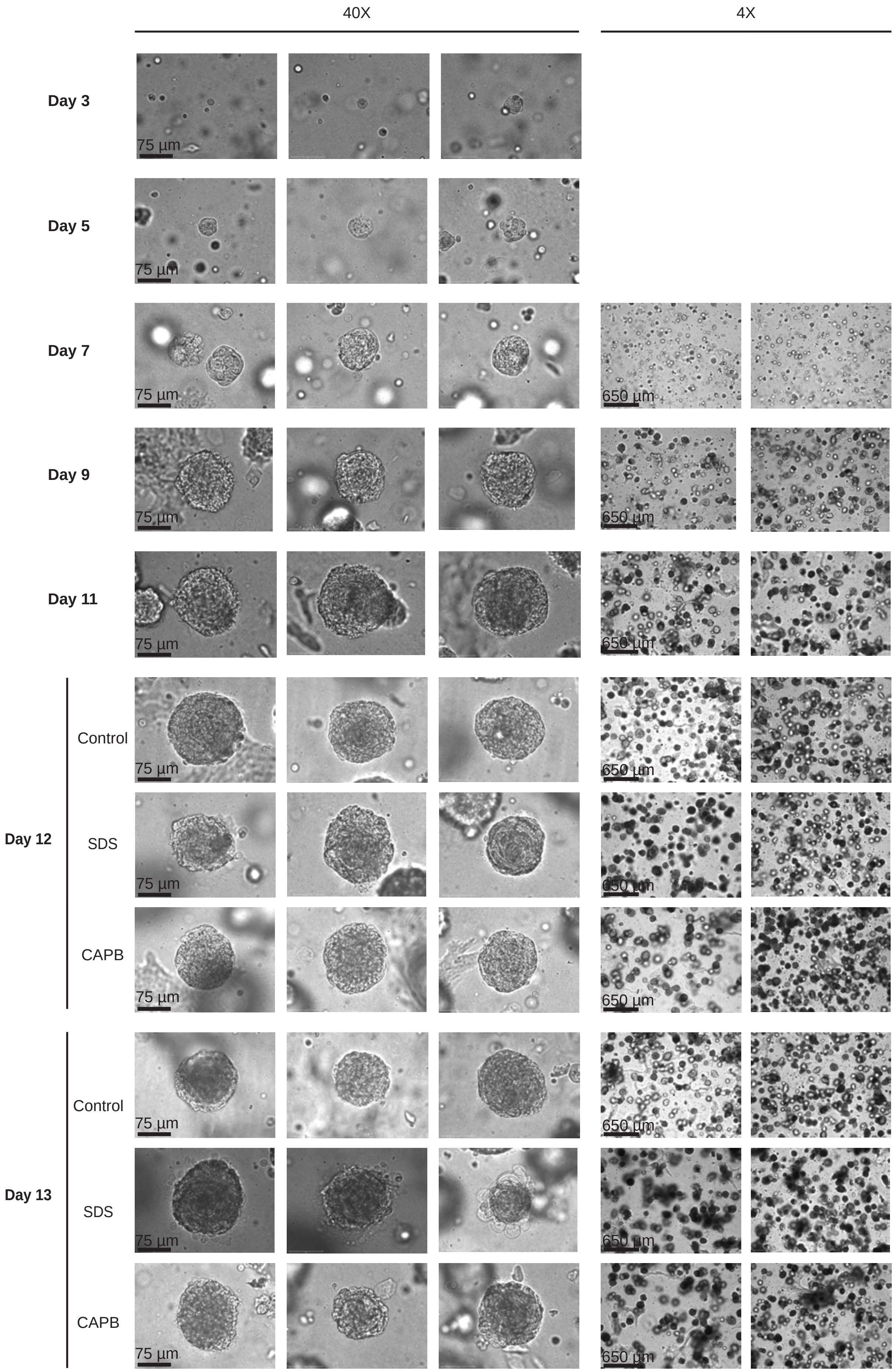

Figure S1. Ardicli et al.
