# Supplementary material for: Pathways of Epithelial Barrier Breakdown and Remodeling in Esophageal Organoids by Commonly Used Surfactants
Source: Allergy. 2026 Aug 27;81(10):3659–72. doi: 10.1111/all.70490 (PMC13646889; doi:10.1111/all.70490)
Supplement: Supplementary file 2 [file ALL-81-3659-s001.docx]

**Table S1.** Baseline demographic, clinical, endoscopic, histopathological, and treatment characteristics of the patients

|  | Donor 1 | Donor 2 | Donor 3 | Donor 4 |
| --- | --- | --- | --- | --- |
| Age | 37 | 72 | 59 | 53 |
| Sex | Male | Female | Male | Female |
| Eos/hpf |  |  |  |  |
| - Distal | 10 | 2 | 0 | 0 |
| - Proximal | 5 | 1 | 0 | 0 |
| Symptoms in the past 7 days |  |  |  |  |
| - Dysphagia for solids | Yes | Yes | Yes | Yes |
| - Vomiting | No | No | No | No |
| - Heartburn | No | No | No | No |
| - Abdominal pain | No | No | No | No |
| - Volume Regurgitation | No | No | No | No |
| - Dysphagia for solids and semisolids | No | No | No | No |
| - Odynophagia | No | No | No | No |
| - FIRE | No | No | No | No |
| Smoking status | Yes | No | No | No |
| White exudates |  |  |  |  |
| - Distal | Absent | Absent | Absent | Absent |
| - Proximal | Absent | Absent | Absent | Absent |
| Furrows |  |  |  |  |
| - Distal | Absent | Absent | Absent | Absent |
| - Proximal | Absent | Absent | Absent | Absent |
| Fixed rings |  |  |  |  |
| - Distal | Absent | Absent | Mild | Mild |
| - Proximal | Absent | Absent | Mild | Absent |
| Stricture |  |  |  |  |
| - Distal | Absent | Absent | Absent | Absent |
| - Proximal | Absent | Absent | Absent | Absent |
| Vascular pattern |  |  |  |  |
| - Distal | Normal | Normal | Normal | Normal |
| - Proximal | Normal | Normal | Normal | Normal |
| PPI treatment | Yes | No | No | Yes |
| Steroids treatment | Yes | Yes | Yes | Yes |

**Table S2.** Information on the primary and secondary antibodies used in confocal microscopy

|  | **Target** | **Fluorochrome** | **Brand** | **Catalog number** | **Dilution** |
| --- | --- | --- | --- | --- | --- |
| **Primary antibodies** |  |  |  |  |  |
|  | Mouse anti-Vimentin | AF546 | Abcam | AB8978 | 1:100 |
|  | Rabbit anti-E-cadherin | AF488 | Cell Signaling Technology | 3199S | 1:200 |
| **Secondary antibodies** |  |  |  |  |  |
|  | Goat anti-rabbit IgG | AF488 | Invitrogen | A11034 | 1:500 |
|  | Goat anti-mouse IgG | AF546 | Invitrogen | A11030 | 1:500 |
|  | Dapi |  | Sigma | 10236276001 | 1:5000 |
